# Meeting the Industrial Challenges of CO_2_ Photocatalytic Reduction: Moving From Molybdenum Disulfides to Oxysulfides Based Materials?

**DOI:** 10.1002/cssc.202400572

**Published:** 2024-09-10

**Authors:** Sébastien Roth, Audrey Bonduelle‐Skrzypczak, Christèle Legens, Pascal Raybaud

**Affiliations:** ^1^ IFP Energies Nouvelles Rond-Point de l'Echangeur de Solaize 69360 Solaize France

**Keywords:** Photocatalysis, Carbon storage, MoS_2_, TiO_2_, Oxysulfides

## Abstract

Reducing CO_2_ emissions is one of the greatest challenges of the century. Among the means employed to tackle CO_2_ emissions, the photocatalytic conversion of CO_2_ is an appealing way to valorize CO_2_ since it uses the sun energy, which is abundant. However, nowadays, the best photocatalytic systems still report too low efficiencies, and use expensive materials, so they cannot be readily industrialized for use at large scale. In this report, we first highlight general industrial and process challenges (including operating conditions). Then, focusing on MoS_2_/TiO_2_ heterojunction systems, we analyze advantages and limitations of such systems and open perspectives on Mo oxysulfides supported on TiO_2_ discussing their potential to reach higher efficiency for CO_2_ photoconversion.

## Graphical abstract

In this perspective, we stress out the multiscale challenges from the industrial process’ scale down to the material′s atomic scale and highlight the great potential of Mo oxysulfides. Hence to meet the industrial challenges of CO_2_ photocatalytic reduction, we propose to move from the MoS_2_/TiO_2_ heterojunction to MoO_x_S_y_/TiO_2_ systems.

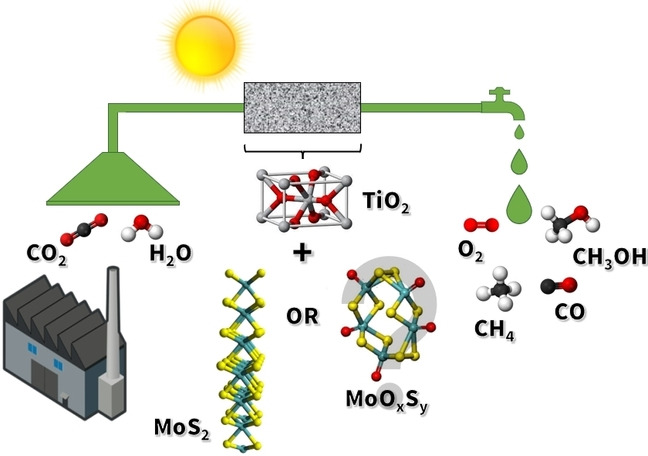

## 1 Introduction

Nowadays, most of the energy requirements are still fulfilled by the combustion of fossil fuels such as coal, oil and natural gas,^[1]^ leading to a continuous increase in the CO_2_ level in the atmosphere. Its current atmospheric concentration (424 ppm in February 2024^[2]^) is higher than at any point in at least the past 800 000 years,^[3]^ resulting in unprecedented global warming. A critical rise in temperature would cause disastrous environmental consequences such as ice melting at the Earth′s poles, collapse of the biodiversity and increases in precipitations, droughts and wildfires.^[4]^ To limit such consequences, a target value of “well below 2 °C above pre‐industrial temperature levels” was set by the Paris Agreement in 2015. This means that CO_2_ emissions need to fall from 36.6 Gt in 2021, to 12 Gt (at maximum) in 2050, following the Announced Pledges Scenario, which would result in a +1.7 °C in 2100.^[1]^

Reducing CO_2_ concentration is hence one of the major challenges of our society. Currently, the main strategies to reduce CO_2_ emissions are: 1) reducing energy consumption 2) decarbonizing the energy sources 3) CO_2_ capture and storage (CCS) or utilization (CCU). The first one, also called “energy sobriety” by governments, can hardly allow to reduce CO_2_ emission by itself, given the expected world population growth and energy demand, but it can help to refrain the increase. The second one translates in the development of renewable sources of energy. But they still represented less than 12 % of the global energy consumption in 2021.^[1]^ In cause, their difficult handling and their lower efficiency compared to nonrenewable sources. The third one aims at reducing CO_2_ emissions by capturing and/or utilizing it directly from the atmosphere, or at the exhaust of plants like cement factories. CCS is a promising technology but its high cost, limited storage capacity and elevated carbon footprint limit its further development.^[5]^ CCU seems then more promising since it converts CO_2_, via biological, physical or chemical/catalytic processes, into value‐added products ranging from fuels and chemicals (like methane, methanol, formic acid, etc.) to microalgae, that can be sold or directly re‐used.[^5^, ^6^]

Among the CO_2_ conversion methods (thermal, electrochemical, biochemical, etc.),[^7^, ^8^] photocatalysis is an appealing one since it exploits the sun energy, which is abundant (400 times higher than the annual energy demand^[9]^). However, the photocatalytic conversion of CO_2_ is a complex process which still suffers from a very low energy efficiency yield, well below the target of 10 % efficiency for at least 10 years,[^10^, ^11^] hence it cannot be readily applied at an industrial scale. The low efficiency arises from many physical and chemical limitations of the materials, such as light penetration and absorption, charges recombination, CO_2_ stability, selectivity, competing hydrogen evolution reaction (HER), etc.^[12]^

This perspective aims at introducing an appealing class of materials for heterogeneous CO_2_ photoreduction with great potential to reach unprecedented energy efficiencies. For this purpose, we first recall the main process and industrial challenges linked to CO_2_ photoreduction. Then, we discuss about TiO_2_, MoS_2_ and their heterojunction as a potential photocatalytic system for industrial applications. Finally, we present the potential of an alternative class of materials for CO_2_ photoreduction: Mo oxysulfides.

## 2 Challenges for Implementing CO_2_ Photoreduction Process at an Industrial Scale

The industrialization of the photocatalytic conversion of CO_2_ could be directly applied to the fumes of cement plants, for instance, since they are mainly composed of water and CO_2_, inevitably derived from the conversion of CaCO_3_ to CaO. However, nowadays the best photocatalysts (like 1 %Pt/TiO_2_ ^[13]^) still record low conversionenergetic efficiencies of about 1 %. Making some simple assumptions: only methane is produced, mean solar irradiance of 270 W/m^2^ (for a desert type of land),^[14]^ no kinetic limitations, no ageing of the photocatalyst, and considering a typical cement factory which produces 75 tons of CO_2_ in 1 hour,^[15]^ it is possible to evaluate the feasibility of the CO_2_ conversion by a photocatalytic process. Such data imply that an irradiated surface of 370 km^2^, using the best photocatalysts currently known, would be needed to totally convert the emitted CO_2_ of a cement plant. This represents 26 Ivanpah solar plants (14 km^2^).^[16]^ If we compare to nature′s ability to convert CO_2_ with photosynthesis, taking 22 kg of CO_2_ absorbed by one tree per year,^[17]^ and a tree density of 125 000 trees per km^2^ (8 m^2^ per tree), we would need a surface of 200 km^2^ covered by trees to totally convert the CO_2_ emitted during 1 year by a cement plant.

As additional limitation, choosing the 1 %Pt/TiO_2_ photocatalyst to convert the CO_2_ emitted by a cement plant would require 94 tons of Pt to synthesize enough catalyst to cover 370 km^2^. This represents about 50 % of the global Pt production.^[18]^ It is hence not a viable photocatalyst to use.

Therefore, for sustainability and feasibility reasons, there is a need to optimize the process and also to develop alternative photocatalysts with higher efficiencies, that do not involve scarce elements such as platinum. Indeed, these two scopes for improvement are key if we want to make the photocatalytic reduction of CO_2_ at an industrial scale a reality.

## 3 Process Optimization for High Efficiency Heterogeneous CO_2_ Photoconversion

### 3.1 Liquid‐Solid Versus Gas‐Solid Systems

Heterogeneous CO_2_ photoconversion can be performed with liquid‐solid and gas‐solid systems. Liquid‐solid photocatalytic systems are generally made of an aqueous dispersion of a photocatalyst nanoparticles. In this configuration, the photocatalyst is more easily accessible for the photons and the reactants. The processing is also easier than for gas‐solid systems. However, the low solubility of CO_2_ in water (34 mmol/L at 25 °C)^[19]^ limits the quantity of CO_2_ adsorbable by the photocatalyst. Additionally, the separation of products from reactants and photocatalytic materials are quite challenging.^[20]^ Finally, in liquid‐solid systems, the catalytic surface is solvated by water which saturates the surface by hydroxyl groups. Those hydroxyls prevent the adsorption and activation of CO_2_ molecules on the Lewis acid sites.^[21]^

In contrast, gas‐solid photocatalytic systems are generally made of an immobilized photocatalyst (supported or not) on which a gas flux or reactants will pass. This approach makes it easier to separate the different compounds and allow different configurations for the reactor: batch or continuous. With such type of systems, the CO_2_/H_2_O ratio can be tuned, the problem of CO_2_ solubility in water being overcome. Adsorption and desorption of reactants and products are also facilitated.^[22]^ However, this approach can decrease the accessible area for photons.

From an industrial point of view, gas‐solid photocatalytic systems are more interesting since one of the ultimate goals of such systems would be, for instance, to convert CO_2_ directly from the chimney of cement factories, which can reach CO_2_ concentrations higher than 10 %. In such a situation, CO_2_ comes out already gaseous and humid, so there is no need to add any reactant to the mix. Also, theoretically, greater CO_2_ conversion yield can be expected for gas‐solid systems since there is no solubility limitation, meaning no restriction on the quantity of CO_2_ that can be sent to the photocatalyst.

### 3.2 Batch Versus Continuous Flow Reactor

Batch reactors are generally pressurized closed vessels in which samples of the reactor composition are extracted at given times to analyze its composition and evaluate the CO_2_ photoconversion. This type of reactor can be applied to both liquid‐solid and gas‐solid photocatalysis. The advantage of such reactors is their easy handling and operating. However, the accumulation of products within the reactor favors their readsorption and hence their reverse or side reaction like the re‐oxidation into CO_2_.^[23]^ They are also not really suitable for long‐time and large‐scale applications. In continuous flow reactors, the reactants and products are moving at a constant flow rate. The advantage of such reactors is that in‐line analyses can be carried out, so it allows a better time dependence following of the reaction. Also, they can avoid the problem of products accumulation and readsorption encountered with batch reactors. However, the contact time of reactants with the photocatalyst is necessarily smaller, which can lead to a decrease of the overall conversion yield.^[12]^

The structure of the reactor also plays a key role on the photocatalytic process. For instance, fiber coated,^[24]^ monolith‐type with honeycomb‐like structure,^[25]^ or well‐organized nanoporous^[26]^ photoreactors were developed to enhance light harvesting. Dual chamber reactor can also be used to separate reduction and oxidation reactions in batch reactors in order to improve the stability of the photocatalyst.^[27]^

Overall, continuous flow reactors should be preferred for industrial applications for all the advantages mentioned previously and also because they can be scaled up more easily.

### 3.3 Operating Conditions

The main parameters that impact the process efficiency are the temperature, partial pressure of CO_2_, mass of photocatalyst, CO_2_/H_2_O (or H_2_) ratio, light intensity, wavelengths range of irradiation, volume of solution or gas (for batch reactors) and gas flow rate (for continuous flow reactors).

#### 3.3.1 Temperature

A temperature increase can have several effects on the photocatalytic system: 1) influence on the rate of reactions since the kinetic rate constants are generally increasing exponentially with temperature 2) decrease of the amount of dissolved CO_2_ in liquid‐solid photocatalysis systems 3) increase of charges recombination probability.^[28]^ Depending on the rate limiting step, increasing the temperature can either favor or disfavor the photocatalytic activity. However, experimental results showed that small temperature variations (26 to 36 °C) do not affect the CO_2_ conversion rate.^[29]^

#### 3.3.2 Pressure and Relative Concentrations

A pressure increase does not influence so much the overall reaction yield, it rather influences the selectivity of the process. In liquid‐solid systems, increasing the CO_2_ pressure favors the production of gaseous products like CH_4_ at the expense of liquid ones like CH_3_OH.^[29]^ In gas‐solid systems, increasing the CO_2_ partial pressure could lead to the formation of longer chains products (C_2_ and C_3_) like ethane and propane.^[30]^ Also, the overall conversion yield increases when CO_2_ partial pressure increases, up to a certain point corresponding to the photocatalyst saturation: when adsorption will not be the kinetically limiting step anymore.^[23]^

If the CO_2_/H_2_O ratio is too high, the CO_2_ conversion is not optimal due to a lack of proton source. However, if this ratio is too low, the impact is negative as well, favoring Hydrogen Evolution Reaction (HER) instead of CO_2_ photoreduction.^[31]^ This ratio is however hard to tune in liquid‐solid state photocatalysis due to the low solubility of CO_2_ in water.

#### 3.3.3 Illumination

The wavelength range of irradiation has to be adapted to the bandgap of the photocatalyst. Moreover, it can remarkably impact the activity and selectivity of materials. For instance, Hezam et al. reported a MoS_2_/TiO_2_ system with switchable CO_2_ reduction products (from CO to CH_4_) when illuminated with solar or visible light.^[32]^

Increasing the light intensity increases the production yield at rather low intensities (the production yield is proportional to the square root of the light intensity) due to an increase of available photons when the absorption is the kinetically limiting step. However, at higher intensities, increasing the light intensity favors charges recombination and hence decreases the overall conversion yield.^[31]^

#### 3.3.4 Catalyst vs Reactant Quantity

The increase of the photocatalyst quantity in liquid‐solid systems improves the overall efficiency up to a certain point when it will start to have a screening effect and prevent some photocatalyst particles to be illuminated. The same situation happens with gas‐solid systems, mainly due to the limited penetration of light inside the material (less than 1 μm for bulk TiO_2_ ^[33]^ and few μm for TiO_2_ thin films^[34]^).

The increase of volume of solution or gas in batch reactors can lead to diffusion or inhomogeneities problems. In continuous flow reactors, the gas flow rate influences the contact time of the reactants with the photocatalyst. Decreasing the flow rate is beneficial for the process activity up to a certain point where a plateau is reached, which means that the reactants contact time is not the rate limiting step anymore.^[35]^

### 3.4 Choice of Reactants

Different reactants can be used in order to photoreduce CO_2_, the most common is water as protons source and holes scavenger, but H_2_ can replace water. Sacrificial agents can also be used as hole scavengers.

The use of sacrificial agents is very common for liquid‐solid photocatalysis. One way to improve the CO_2_ photoconversion yield is to replace water by another solvent in which CO_2_ solubility is greater. In this case, sacrificial agents are needed for the oxidation reaction. They can also be used in combination with water solvent due to their stronger hole accepting property. Classical sacrificial agents are salts (Na_2_S/Na_2_SO_3_), acids (lactic acid, ascorbic acid), alcohols (methanol, ethanol, phenols) or tertiary amines (TEOA, TEA).^[20]^ The use of methanol as sacrificial agent is controversial because it raises the problem of distinguishing methanol used as sacrificial agent from methanol photoproduced from CO_2_ reduction.^[36]^ In any case, it is better to avoid the use of sacrificial agents from an economic point of view for industrial processes. Indeed, they are more or less expensive consumables that are not recovered after the reaction.

For gas‐solid photocatalysis, H_2_ can be used to replace H_2_O or as a co‐reactant. The CO_2_/H_2_ mix can result in the same CO_2_ photoreduction products as for the CO_2_/H_2_O mix: CH_4_ and CO were obtained on oxide photocatalysts (NiO, Fe_3_O_4_, CuO),^[37]^ CH_3_OH and CO on layered double hydroxide^[38]^ or surface frustrated Lewis pairs^[39]^ systems. Even CO_2_/(H_2_O + H_2_) mix can be used and proved to be the best mix compared to CO_2_/H_2_ or CO_2_/H_2_O on TiO_2_.^[40]^ Changing the reactants mix also influences the selectivity of the photoreaction probably owing to different reduction mechanism pathways.^[41]^ Despite the CO_2_/H_2_O mix generally shows better results than CO_2_/H_2_ for the same photocatalytic system,[^41^, ^42^, ^43^] the advantage to use H_2_ is to have a better control over the reactants quantities and to avoid the  competing HER. Moreover, using H_2_ as a reactant makes also sense from an industrial point of view. Indeed, the fumes coming out of chimneys can be treated to condense water and produce H_2_ with the help of an electrolyzer. The so produced H_2_ would then be used for the photocatalytic reaction.

## 4 Search for Alternative Materials for High Efficiency Heterogeneous CO_2_ Photoconversion

**Scheme 1:**
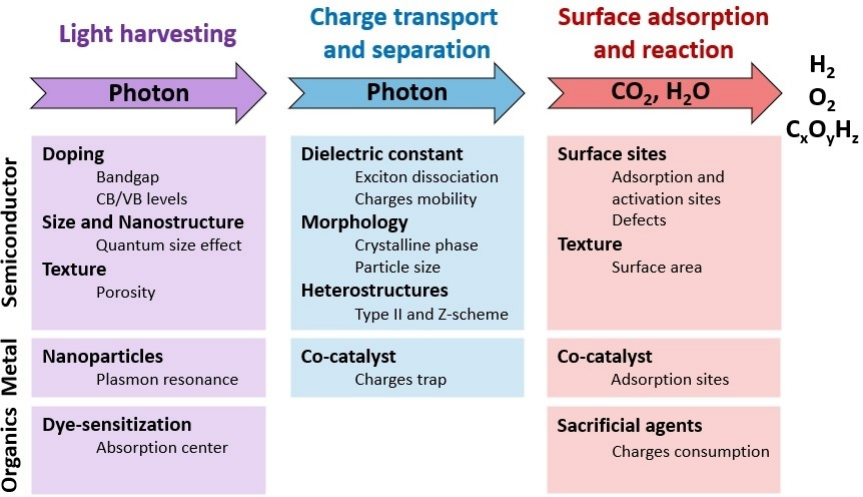
Summary of the photocatalyst design strategies for enhancing CO_2_ photoconversion efficiency.

To tune the materials physical and chemical properties and enhance the overall photoactivity is a huge challenge that has already been widely described in literature.[^44^, ^45^, ^46^] Scheme 1 summarizes the different materials design strategies that can be applied to try to improve each step of the photocatalytic mechanism: 1) light penetration and absorption, 2) charge transport and separation, 3) reactants surface adsorption and redox reactions.

For the first step improvement, the semiconductor texture, size and dopants are the key features. Additionally, metallic nanoparticles involving plasmon resonance, and/or dyes can be deposited on the photocatalyst so as to improve light harvesting. For the second step, the morphology and dielectric constant of the material are important parameters but the main strategy employed to improve charges transport and separation is to combine a semiconductor with another one (to form heterojunction schemes), or with a co‐catalyst. Finally, for the third step, the main strategies concern the texture and surface sites of the semiconductor. Metallic dopants can also provide active sites, and the use of sacrificial agent is popular to avoid limitations on the oxidation part.

One should not forget that one strategy can impact several steps of the photocatalytic process, even if only the most impacted step was aforementioned for simplicity.

Hence, the goal of this part is not to describe in detail the design of photocatalysts to improve their efficiency, but it is rather to introduce a new class of materials with high potential for CO_2_ photoconversion. We will predominantly focus on the challenges for the CO_2_ reduction reaction, and address more occasionally (whenever it is required) some linked to the Oxygen Evolution Reaction (OER).

### 4.1 TiO_2_: A Reference Photocatalyst

TiO_2_ is a well‐known semiconductor and the Degussa TiO_2_ (P25), which is a mix of anatase (80 %) and rutile (20 %) is still considered as a model photocatalyst.^[47]^ It is not the aim of the present report to review this reference material which has been widely studied in literature. In spite of its chemical stability, long durability, non‐toxicity, low cost and easy availability, it is far from being the ideal photocatalyst. Indeed, its conversion efficiency for CO_2_ photoreduction is low due to many factors: 1) its wide bandgap (3.0–3.2 eV) that allows only UV‐light absorption (5 % of the total sun spectrum), 2) its conduction band (CB) energy position (−0.5 V vs NHE at pH=7)^[20]^ that does not allow a huge overpotential with the reactions of interest (CH_4_,CO,…/CO_2_≤−0.24 V vs NHE at pH=7), 3) its high electrical resistivity (10^13^–10^18^ Ω.cm) and 4) a limited quantity of adsorption sites due to its small surface area in general (~50 m^2^/g for TiO_2_ P25). Another downside of TiO_2_ is that the OER, which is the necessary counter reaction in the CO_2_ photoreduction, is not very often analyzed in the literature. Indeed, the absence of the observed O_2_ product can be related to several reasons: 1) kinetic limitations in H_2_O oxidation, 2) the adsorption of produced O_2_ in TiO_2_ oxygen vacancies, and 3) the consumption of produced O_2_ or O‐containing species for backward reactions (CH_4_ oxidation).^[34]^

Many strategies are reported in literature to improve TiO_2_ photoactivity for CO_2_ conversion: doping,^[48]^ nanostructuration,^[49]^ co‐catalysts,^[13]^ heterojunction with another semiconductor,[^50^, ^51^] etc. A non‐exhaustive summary of TiO_2_ based photocatalysts for CO_2_ conversion is reported in Table 1. Reported values for produced and used electrons for CO_2_ conversion (in e^−^ μmol/h/g) were derived from the products rate of formation (in μmol/h/g) and the number of electrons required to form such products.

**Table 1:** TiO_2_ based photocatalysts for CO_2_ conversion: operating conditions, produced and used electrons for CO_2_ conversion expressed in e‐ μmol/h/g, gain compared to the reference TiO_2_ of the corresponding work.

Material	Conditions	Produced and used electrons for CO_2_ conversion (e‐ μmol/h/g)	Gain compared to reference TiO_2_
TiO_2_ (anatase)^[54]^	Solid‐gas, continuous flow reactor, CO_2_+H_2_O	1.3	‐
TiO_2_ (rutile)^[54]^	Solid‐gas, continuous flow reactor, CO_2_+H_2_O	0.4	‐
TiO_2_ P25 (20 % rutile, 80 % anatase)^[49]^	Solid‐gas, continuous flow reactor, CO_2_+H_2_O	2.3	‐
N‐doped TiO_2_ (anatase)^[48]^	Solid‐gas, batch reactor, CO_2+_H_2_O	31	x2
TiO_2‐x_ (101) (anatase)^[55]^	Solid‐gas, continuous flow reactor, CO_2_+H_2_O	16	x2
3D‐TiO_2_@Si foam (10 % rutile, 90 % anatase)^[49]^	Solid‐gas, continuous flow reactor, CO_2_+H_2_O	9.9	x5
Au/TiO_2_ (anatase)^[13]^	Solid‐gas, continuous flow reactor, CO_2_+H_2_O	56.8	x7
Pt‐TiO_2_ UV100 (anatase)^[13]^	Solid‐gas, continuous flow reactor, CO_2_+H_2_O	720	x90
Type II co‐exposed (101) and (001) TiO_2_ (anatase)^[56]^	Solid‐gas, batch reactor, CO_2+_H_2_O	10.8	x9
Type II Cu_2_ZnSnS_4_/TiO_2_ (anatase)^[50]^	Solid‐gas, batch reactor, CO_2+_H_2_O	14.6	x12
S‐scheme Zn_3_In_2_S_6_/TiO_2_ (rutile and anatase mix)^[51]^	Solid‐gas, batch reactor, CO_2+_H_2_O	96.2	x50
Au@CdS/TiO_2_ (Inverse Opal) (20 % rutile, 80 % anatase)^[57]^	Solid‐gas, continuous flow reactor, CO_2_+H_2_O	334	x24

As shown in Table 1, the best gains in activity are obtained when TiO_2_ is combined with another materials: a co‐catalyst or another semiconductor. Actually, the best performances reported are for the Pt‐TiO_2_ UV100 catalyst, which uses Pt nanoparticles as co‐catalyst and electrons trap, supported on a very high specific surface TiO_2_ UV100 (400 m^2^/g).^[49]^ This highlights the need to combine TiO_2_ with another material in order to tackle some of its previously mentioned limitations. In this regard, MoS_2_ seems to be a good candidate since it possesses a small bandgap and high electronic conductivity.

### 4.2 MoS_2_: An Excellent Electrocatalyst, but Not Only

MoS_2_ is a visible light responsive photocatalyst that is made of earth‐abundant elements with promising properties. It can be easily doped, and its bandgap (1.29 – 1.90 eV) can be tuned depending on the number of layers, the surface defects, the temperature and/or the strain applied to the material.^[52]^ However, improvements need to be done essentially on two points: 1) its photostability: MoS_2_ gets easily oxidized by photogenerated holes during the photocatalytic process, 2) its small bandgap that limits its range of applications as a photocatalyst. Actually, MoS_2_ (2H and/or 1T) is often used for its co‐catalyst electrons trap property, but for the 2H phase, its semiconductor properties can be exploited as well.

**Scheme 2:**
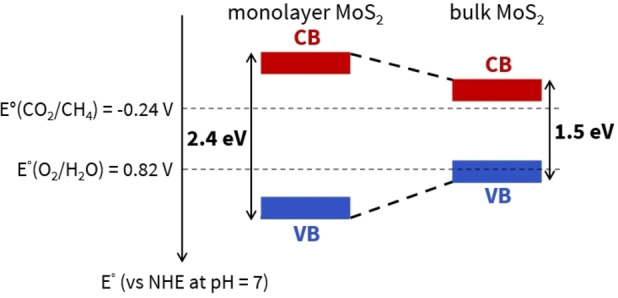
Monolayer MoS_2_ and bulk MoS_2_ valence band (VB) and conduction band (CB) positions for CO_2_ photoreduction. The CO_2_/CH_4_ redox couple is the one with the highest potential for CO_2_ reduction. Bandgaps value reported here were extracted from DFT calculations.^[58]^ These values tend to be overestimated compared to experimental values, due to the fact that DFT calculations evaluate the fundamental bandgap, while experimental studies often determine the optical bandgap.^[60]^ Note also that experimental values vary depending on the characterization techniques used.[^61^, ^62^]

Asadi et al. were the first ones to report the use of MoS_2_ for CO_2_ electroreduction in 2014.^[53]^ They showed that MoS_2_ gives better results than noble metals in CO_2_ electroreduction and that the activity is mainly due to MoS_2_ edges. Indeed, vertically aligned MoS_2_ on glassy carbon showed higher current density than bulk MoS_2_. This initial study paved the way to the research in the field of MoS_2_ based systems for CO_2_ photoreduction. However, the band positions of MoS_2_, which depend on its number of layers, are not optimal to favor CO_2_ photoreduction as well as OER, even for MoS_2_ single layer which shows the widest bandgap (2.4 eV) (Scheme 2).^[58]^ Indeed, large overpotentials (at least 0.7 V) on both the reduction and the oxidation sides may be required from a kinetic point of view^[59]^ and it is still an open question to which extent the VB and CB band positions of MoS_2_ single layer can be compatible with the potentials required for both the reduction and oxidation reactions.

Hence, as reported in Table 2, several strategies have been developed to make MoS_2_ based materials, potential candidates for CO_2_ photoreduction reaction and OER, mainly using MoS_2_ with either metallic co‐catalysts like Ag and Au^[63]^, or wide bandgap semiconductors like TiO_2_,^[64]^ SiC^[22]^ and g−C_3_N_4_,^[65]^ or involving two materials in heterojunction.[^66^, ^67^] The best systems however use three different materials in heterojunction[^66^, ^67^] leading to better charges separation and band positions adjustment.

**Table 2:** MoS_2_ based photocatalysts for CO_2_ conversion: operating conditions, produced and used electrons for CO_2_ conversion expressed in e‐ μmol/h/g, gain compared to the reference photocatalyst of the corresponding work.

Material	Conditions	Produced and used electrons for CO_2_ conversion (e‐ μmol/h/g)	Gain compared to reference material
2H‐MoS_2_ ^[64]^	Solid‐liquid, batch reactor, CO_2_+H_2_O	15.7	‐
(2H+3R)‐MoS_2_ ^[68]^	Solid‐liquid, batch reactor, CO_2_+H_2_O	94.8	‐
Ag/MoS_2_ ^[63]^	Solid‐liquid, batch reactor, CO_2_+H_2_O	31	x2.4 (vs MoS_2_)
Au/MoS_2_ ^[63]^	Solid‐liquid, batch reactor, CO_2_+H_2_O	16	x2.6 (vs MoS_2_)
Type II TiO_2_/MoS_2_ ^[64]^	Solid‐liquid, batch reactor, CO_2_+H_2_O	156.2	x10 (vs MoS_2_) x7 (vs TiO_2_)
S‐scheme SiC (3D)/MoS_2_ (2D)^[22]^	Solid‐gas, batch reactor, CO_2_+H_2_O	105.2	x30 (vs MoS_2_) x4 (vs SiC)
S‐scheme g‐C_3_N_4_/MoS_2_ ^[65]^	Solid‐liquid, batch reactor, CO_2+_H_2_O	17.1	x3 (vs g‐C_3_N_4_)
TiO_2_‐rGO‐MoS_2_ ^[66]^	Solid‐gas, batch reactor, CO_2+_H_2_O	186.4	x14.5 (vs TiO_2_)
SnS_2_‐rGO‐MoS_2_ ^[67]^	Solid‐liquid, batch reactor, CO_2+_H_2_O	451.2	x205 (vs MoS_2_)

### 4.3 The MoS_2_/TiO_2_ Heterojunction: An Inspiration for Photocatalysts

The MoS_2_/TiO_2_ heterojunction is a particularly interesting system for CO_2_ photoreduction, even if it is well‐known that Pt/TiO_2_ is one of the best photocatalysts for that reaction. Indeed, it has been shown that MoS_2_ as a co‐catalyst can challenge Pt for the hydrogen evolution reaction when combined to TiO_2_.[^69^, ^70^] It is hence interesting to study how this type of material behaves for CO_2_ photoreduction.

**Figure 1:**
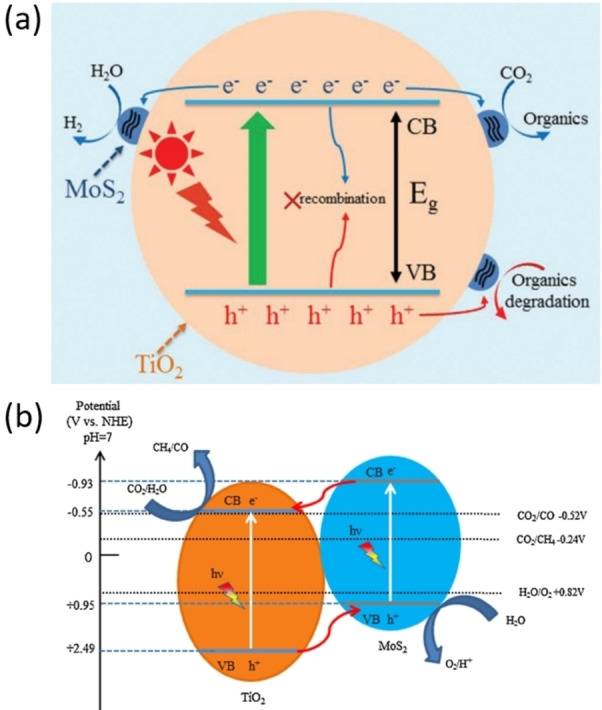
(a) MoS_2_ co‐catalyst of TiO_2_ for CO_2_ photoconversion and organics photodegradation. Reproduced with permission from Ref. [71]. Copyright 2017, RSC Pub. (b) Type II heterojunction MoS_2_/TiO_2_ for CO_2_ photoconversion. Reproduced with permission from Ref. [64]. Copyright 2019, Elsevier.

In experimental studies, the TiO_2_/MoS_2_ heterojunction has been mainly reported in two categories. The first and most common one reports only the role of MoS_2_ as a co‐catalyst, while TiO_2_ harvests the light.^[71]^ Therefore, the role of MoS_2_ is to act as electrons trap in order to limit charges recombination, and to provide supplementary active sites (Figure 1.a). In this case, the metallic property of 1T‐MoS_2_ is exploited as a co‐catalyst since it has better charge conductivity, or because 2H‐MoS_2_ band edges are not adequate for the redox reactions.^[72]^ The second category invokes the possible formation of a type II heterojunction or of a Z‐scheme. In the former case, the charge transfer mechanism will result in the accumulation of electrons in the CB of TiO_2_ and of holes in the VB of MoS_2_. So that TiO_2_ takes charge of the reduction reactions and MoS_2_ of the oxidation reactions (Figure 1.b).^[64]^ However, the not negative enough position of the CB of TiO_2_ and the not positive enough position of the VB of MoS_2_ make this type II heterojunction impossible to reach the expected reduction and oxidation overpotentials for high activity.

**Figure 2:**
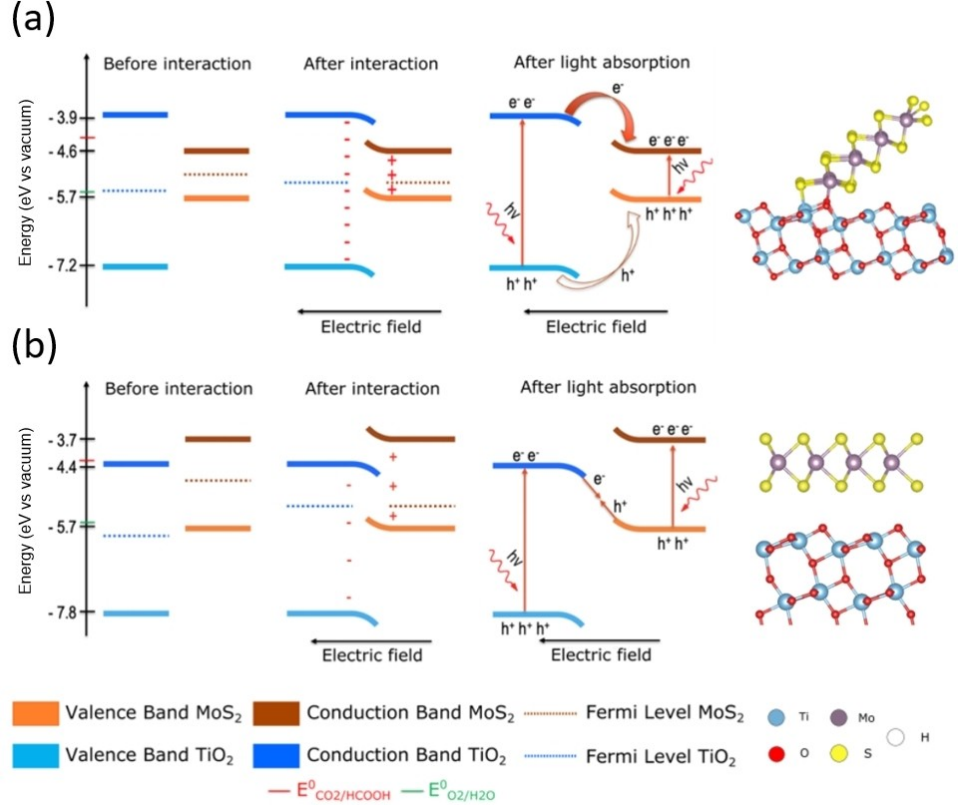
(a) Type I heterojunction of the 1D chemical interface between MoS_2_ nanoribbon and the TiO_2_ (101) surface and associated molecular model. (b) Possible Z‐scheme for the physical 2D‐heterojunction between the MoS_2_ sheet and the TiO_2_ (101) surface and associated molecular model. Adapted with permission from Ref. [73]. Copyright 2022, RSC Pub.

Using density functional theory (DFT) with the proper exchange‐correlation functional (HSE06) to describe bandgaps, Favre et al. investigated various types of MoS_2_/TiO_2_ heterojunction. They distinguished a physical from a chemical interaction between 2H‐MoS_2_ monolayer and anatase TiO_2_ (101) and (001) surfaces, and simulated different possible heterostructures.^[73]^ They showed that a chemical interaction involving Ti−O−Mo interfacial bridges leads to a type I heterojunction due to the band positions of 2H‐MoS_2_ nanoribbons and TiO_2_ (101) surface (which is the major facet in anatase (90 %)). This could not result in improved photocatalytic performances since photogenerated charges accumulate on MoS_2_ which has weaker redox potentials (Figure 2.a). However, a physical (van der Waals) interaction between 2H‐MoS_2_ monolayer and TiO_2_ (101) or (001) surfaces results in a staggered band position. From this point both classical type II heterojunction and Z‐scheme could be considered, even if further calculations of charges distribution at the interface suggest that the Z‐scheme heterojunction could be preferentially formed in this system. It means that electrons accumulate in the CB of MoS_2_ (the strongest reduction potential) and holes in the VB of TiO_2_ (the strongest oxidation potential) (Figure 2.b). Note that such type of Z‐scheme has been often invoked as being crucial for obtaining efficient photocatalysts in CO_2_ reduction^[74]^. The same DFT study also shows that these trends highly depend on the hydroxylated and sulfided states of the TiO_2_ surfaces. In the case of a sulfided surface, the TiO_2_ bandgap is significantly reduced due to a valence band maximum (VBM) shift at higher energy level. Another DFT study completed these results and proposed a Z‐scheme heterojunction also for the MoS_2_/TiO_2_ (100) heterostructure with physical interactions,^[75]^ although the level of theory (GGA) is not accurate enough to guarantee the reliability of the electronic properties. However, classical type II heterojunction could happen if the kinetic effect does not allow the electron migration mechanisms as suggested in Figure 2.b.

The TiO_2_/MoS_2_ heterojunction is mostly reported in literature for pollutants degradation and hydrogen photoproduction.[^71^, ^76^, ^77^] However, there are very few examples of such system for CO_2_ photoconversion. Asadi et al. explained that unsaturated S atoms on MoS_2_ edges are favorable for the HER, and hence for water splitting, while unsaturated Mo atoms exposed on the edge favor the CO_2_ photoreduction.^[53]^ However, it is challenging to synthesize MoS_2_ with mostly Mo atoms on the edges, so this is probably why the use of TiO_2_/MoS_2_ and more generally MoS_2_ for CO_2_ photoconversion is not as widespread as for hydrogen photoproduction or pollutants degradation.

The first report on a TiO_2_/MoS_2_ heterojunction system for CO_2_ photoconversion was published by Tu et al.^[78]^ They prepared a MoS_2_/TiO_2_ nanosheets hybrid that shows excellent liquid‐phase photoconversion of CO_2_ into CH_3_OH. Their best hybrid material is composed of 0.5 wt %MoS_2_ and manifests a 3‐fold in activity compared to TiO_2_ alone (Table 3). This result is due to effective charges separation with electron transfer from the CB of TiO_2_ to the CB of MoS_2_. More loaded samples showed worst results probably because of the light shielding effect of black MoS_2_ nanosheets.

**Table 3:** MoS_2_/TiO_2_ based photocatalysts for CO_2_ conversion: operating conditions, produced and used electrons for CO_2_ conversion expressed in e‐ μmol/h/g, gain compared to the reference photocatalysts of the corresponding work.

Material	Conditions	Produced and used electrons for CO_2_ conversion (e‐ μmol/h/g)	Gain compared to reference material
0.5wt %MoS_2_/TiO_2_ nanosheets^[78]^	Solid‐liquid, batch reactor, CO_2_+H_2_O	63.6	x3 (vs TiO_2_)
8wt %MoS_2_/TiO_2_ nanofibers^[79]^	Solid‐gas, batch reactor, CO_2_+H_2_O	38.2	x9 (vs TiO_2_)
10wt %MoS_2_/TiO_2_ composite^[64]^	Solid‐liquid, batch reactor, CO_2_+H_2_O	156.2	x10 (vs MoS_2_) x7 (vs TiO_2_)
50wt %MoS_2_ flowers/TiO_2_ nanofibers^[81]^	Solid‐liquid, batch reactor, CO_2_+H_2_O	43.2	x2 (vs TiO_2_)
82wt %MoS_2_/TiO_2_ nanowires^[32]^	Solid‐gas, batch reactor, CO_2_+H_2_O	163.2 (visible) 72 (solar)	x45 (vs MoS_2_, visible) x4 (vs MoS_2_, solar)

A MoS_2_/TiO_2_ nanofibers photocatalyst for gas‐phase CO_2_ conversion was then proposed by Xu et al.^[79]^ They observed the formation of both CH_4_ and CH_3_OH while only CH_3_OH was observed on TiO_2_ alone. Their 8 wt % MoS_2_/TiO_2_ exhibits a CO_2_ conversion rate 9 times higher than TiO_2_ alone (Table 3). This improvement is attributed to the increased light absorption thanks to MoS_2_ small bandgap, increased specific surface area, and enhanced charges separation. Similarly, Yu et al. reported a core@shell 3D‐TiO_2_@MoS_2_ heterojunction system for CO_2_ electroconversion.^[80]^ Despite not reporting photocatalytic tests, they showed that their material possessed Ti−S bonds that favor the CO_2_ conversion to CO at the expense of the HER. This is an interesting result that could be extrapolated to CO_2_ photocatalysis.

Jia et al. reported that a MoS_2_/TiO_2_ composite system with 10 wt % MoS_2_ converts CO_2_ into CO and CH_4_, in a liquid‐phase set‐up, with an activity enhanced by a factor 7 and 10 compared to TiO_2_ and MoS_2_ alone respectively^[64]^ (Table 3). These results were assigned to the improved absorption and to lower charges recombination rate due to the type II heterojunction that was formed.

Then, an heterojunction between MoS_2_ flowers and TiO_2_ nanosheets allowed the formation of CO and CH_4_ with a conversion rate that is multiplied by 2 compared to TiO_2_ alone (Table 3) according to Kang et al.^[81]^ They went even further by adding g−C_3_N_4_ to form a multiple heterojunction system that exhibits an even higher conversion rate.

**Figure 3:**
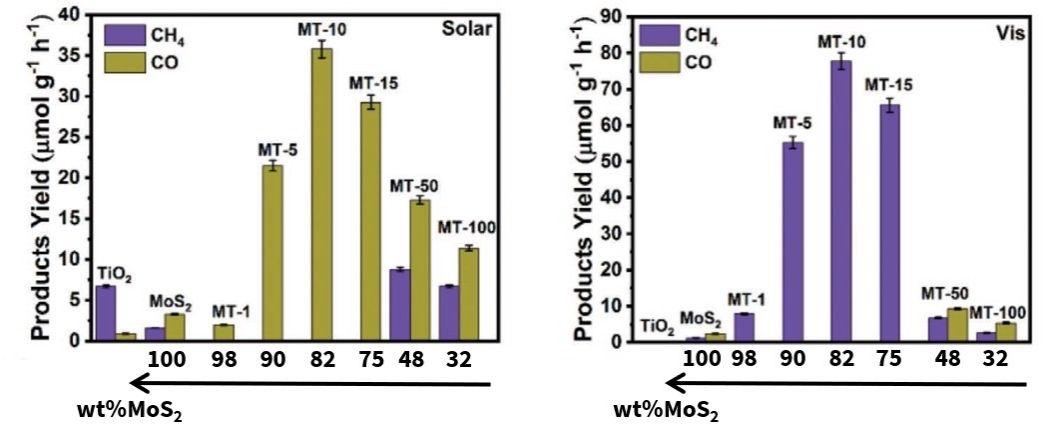
Products yield resulting from CO_2_ photoreduction under visible and solar light. MT−X stands for the experimental conditions which resulted in a given MoS_2_/TiO_2_ heterojunction (M stands for Mo‐containing solution, T stands for Ti‐containing solution, and X is linked to the volume of the Ti‐containing solution used). Adapted with permission from Ref. [32]. Copyright 2023, Wiley‐VCH.

More recently, Hezam et al. reported a MoS_2_/TiO_2_ nanowires system with switchable CO_2_ reduction products (Figure 3).^[32]^ Indeed, they interestingly showed, thanks to in situ irradiated XPS, electron spin resonance, terephthalic acid photoluminescence and photocurrent experiments, that under solar light irradiation, the system exhibits a Z‐scheme type of heterojunction as it was proposed in Figure 2.b.^[73]^ However, under visible light irradiation, it exhibits a type II heterojunction. This change of charges transfer mechanism allowed to switch from CO to CH_4_ respectively as main product of the CO_2_ photoreduction. The switch was ascribed to the change from MoS_2_ to TiO_2_ for the CO_2_ reduction sites. Such system presents an activity (in electrons produced and used for CO_2_ conversion) that is similar to TiO_2_ and up to 4 times higher than MoS_2_ under solar irradiation for the best material. The gain appears to be much more consequent under visible light: up to a 45‐fold in activity compared to MoS_2_ is reported (TiO_2_ does not absorb visible light).

### 4.4 Mo Oxysulfides as Alternative Class of Materials with High Efficiency Potential

A metal oxysulfide is a compound composed of at least a metal, an oxygen and a sulfur atom, with negative oxidation states for both oxygen and sulfur. The classical oxysulfide is a ternary system with the formula M_x_O_y_S_z_, but quaternary and penternary compounds exist as well. Distinction should be made between metal oxysulfides which contain no O−S bond due to the negative oxidation state of S, and metal sulfates where sulfur has a positive oxidation state and binds to oxygen.^[82]^

Up to 1947, ternary oxysulfides were limited to lanthanides, actinides, and bismuth.[^83^, ^84^, ^85^] Oxysulfides were then extended to transition metals like Cu, Zn, Mo, Ti and W. Inoue et al. were the first to report the crystalline structure of two transition metal oxysulfides: MoO_2.74_S_0.12_ and MoO_1.88_S_0.15_.^[86]^ Then Abraham et al. and Pasquariello et al. synthesized various MoO_y_S_z_ amorphous compounds that were used for batteries.[^87^, ^88^] Actually, before being used for photocatalysis applications, metal oxysulfides were primarily used for batteries, scintillators, screens and lasers.^[82]^ They were also reported very early as key intermediates formed during the genesis of industrial hydrotreating supported catalysts. In the last decade, the structure of such amorphous MoO_y_S_z_ intermediates supported on alumina was elucidated both theoretically with DFT simulations,^[89]^ and experimentally by X‐ray absorption spectroscopy.[^90^, ^91^]

Moreover, metal oxysulfide compounds are interesting materials for photocatalysis. Indeed, oxide materials present a too wide bandgap, whereas sulfide materials often present too narrow bandgaps and instability during the photocatalytic process is suspected. In particular, the instability with respect to oxidation (photocorrosion) has been stressed out by numerous studies for the reference CdS photocatalyst[^92^, ^93^] and suspected for MoS_2_.^[94]^ However, this effect is less addressed for oxysulfides. On the one hand, oxysulfides present tunable bandgaps depending on the O and S contents, the more S the narrower the bandgap.^[95]^ On the other hand, the nature of oxysulfides may increase their intrinsic resilience to oxidation due to the S_3p_−O_2p_ hybridization.^[96]^ At this stage, the preparation way of the oxysulfide material may be crucial (under oxidizing environment and specific thermal treatment), since it may induce the formation of stable S‐species and prevent them from a reoxidation process under photocatalytic conditions. However, the complete suppression of sulfur ions oxidation is an open challenge in photocatalysis, which may be limited if holes are efficiently extracted from the oxysulfide phase, either through holes migration to another phase (heterojunction with another semiconductor or coupled to a co‐catalyst), or by improving the oxidation overpotential to favor the OER at the expense of self‐oxidation.

**Figure 4:**
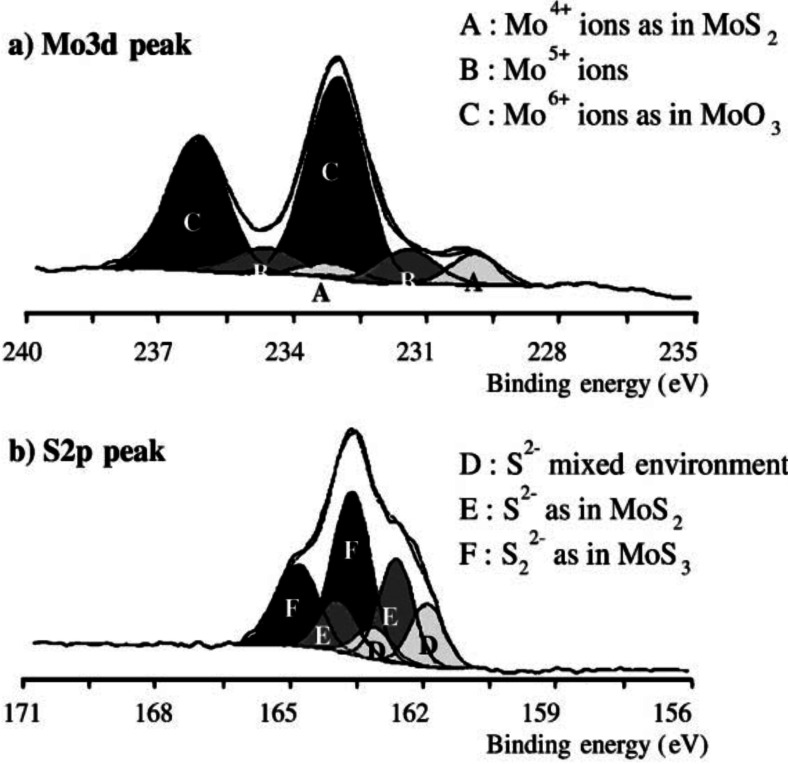
XPS spectra of MoO_2.4_S_0.7_ thin film: (a) Mo 3d_5/2–3/2_ peak; (b) S 2p_3/2–1/2_ peak. Reproduced with permission from Ref. [97]. Copyright 2001, Elsevier.

The intermediate oxidation degree of an oxysulfide, between an oxide and a sulfide, can be revealed by XPS, which reports the presence of typical oxide and sulfide components, as well as a mixed intermediate component. For instance, MoO_2.4_S_0.7_ shows a Mo^6+^ component (oxide‐like), a Mo^4+^ component (sulfide‐like), and a Mo^5+^ component (oxysulfide‐like) (Figure 4.a)^[97]^. This diversity of species is also found for the S_2p_ peak (Figure 4.b). The proportion of each component directly depends on the oxysulfide.

**Figure 5:**
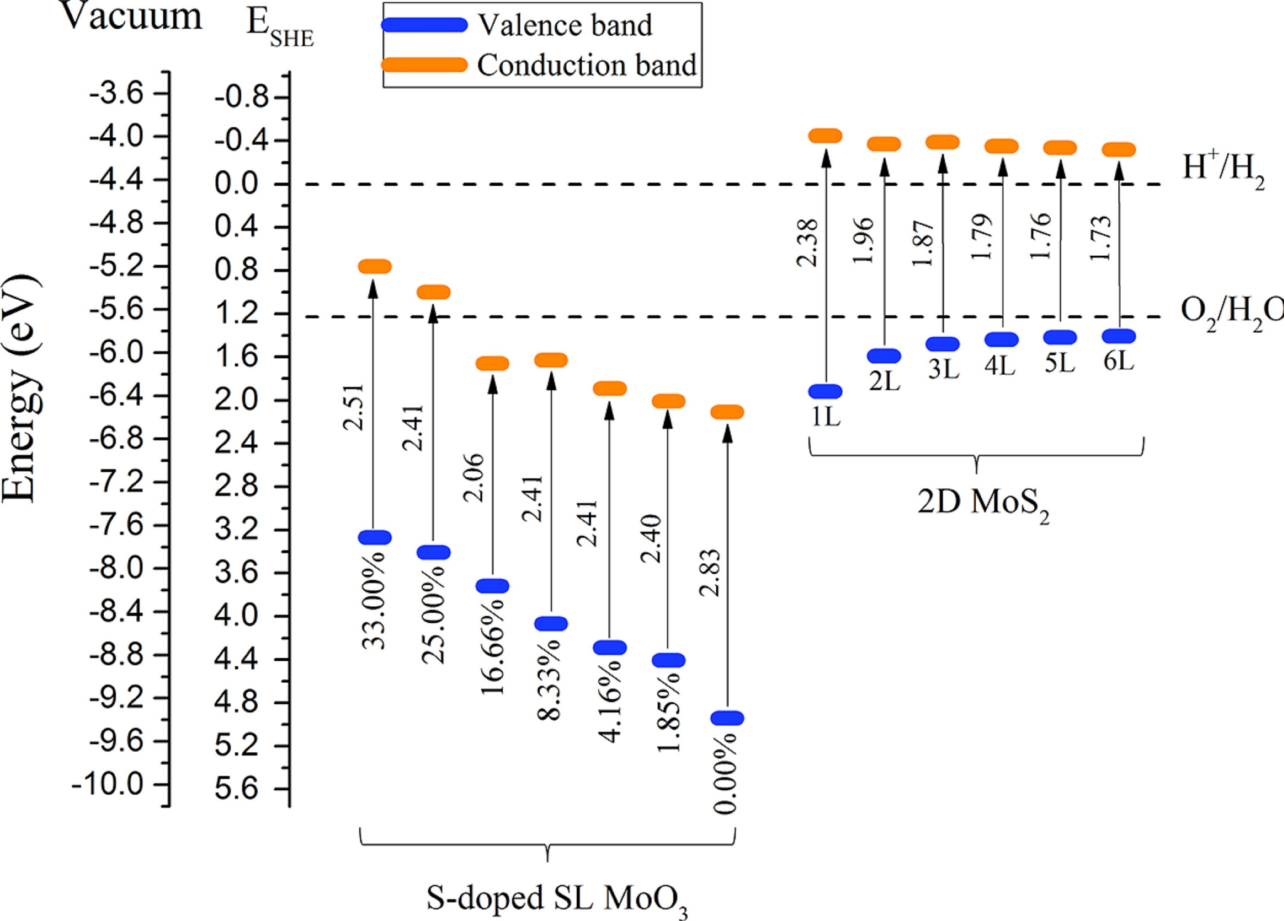
Calculated conduction and valence band edges position for S‐substituted single layer (SL) MoO_3_ and single layer to six layers (1L‐6L) MoS_2_ with respect to the vacuum level and the standard hydrogen electrode at pH=0. Reproduced with permission from Ref. [58], Copyright 2021, American Chemical Society.

Focusing on molybdenum oxysulfides, Shahrokhi et al. conducted DFT calculations of the impact of S‐doping on bulk and few layers MoO_3_ and O‐doping on bulk and few layers MoS_2_, on the opto‐electronic properties of the oxysulfide resulting materials.[^58^, ^98^, ^99^] First of all, they showed that the O doping does not impact so strongly many optical properties of bulk MoS_2_ such as bandgap, absorption coefficient, dielectric constant or even exciton binding energies.^[99]^ This result may imply that if a loss of efficiency of MoS_2_ is observed in oxidizing environment, this is not due to a change of optical properties but rather to a poisoning of surface active sites. By contrast, they showed that the S‐doping of bulk MoO_3_ reduces significantly the bandgap by ~1 eV and increases the optical absorption with respect to pristine α‐MoO_3_, while keeping charge mobility and separation at a level compatible for photocatalysis^[99]^. In addition, the CB and VB edge positions are very sensitive to S‐doping of MoO_3_ and evolves rather continuously to lower potential values (Figure 5). These Mo oxysulfides (S‐doped MoO_3_) exhibit a rather strongly positive VB potential which may be compatible with OER, but the CB band level is not compatible with the negative potential required for CO_2_ reduction. As a consequence, they proposed to build a Z‐scheme heterojunction between S‐doped MoO_3_ (as single layer or multi‐layer) and single layer MoS_2_, which might be suitable for CO_2_ photoconversion.[^58^, ^98^] As illustrated in Figure 5, when the MoO_3_ heterostructure is doped by at least 8 % S, the system exhibits staggered band positions, with bandgaps of similar values ~2.4 eV, characterizing a type II heterojunction or eventually Z‐scheme.

Mo oxysulfides, apart from their opto‐electronic properties, present also interest for orienting the selectivity of reactions. Indeed, Mayhall et al. calculated that water adsorption happen on Mo atoms and is more favorably adsorbed on Mo oxide species than on Mo sulfides.^[100]^ They also reported that water dissociation is favored on Mo oxides. So, the use of oxysulfides for CO_2_ photoconversion is interesting from an opto‐electronic point of view, but also for the reaction selectivity since oxysulfides have greater chances to favor the CO_2_ reduction compared to the HER, oppositely to pure oxide materials.

**Figure 6:**
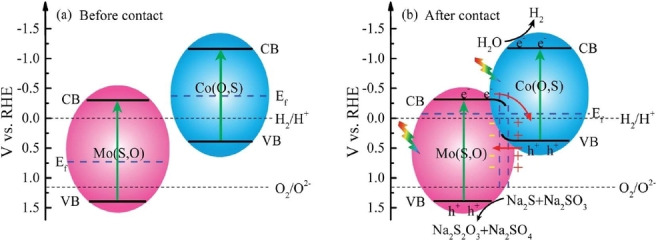
Band positions of the Mo(O,S)/Co(O,S) S‐scheme heterojunction (a) before contact and (b) after contact. Reproduced with permission from Ref. [101], Copyright 2022, RSC Pub.

Transition metal oxysulfides containing Mo have been reported in literature for numerous photocatalytic applications, but never for CO_2_ photoconversion. Indeed, MoCoOS,^[101]^ MoO_y_S_z_‐CoP,^[102]^ MoO_y_S_z_/CdS,^[103]^ and MoO_y_S_z_/Ni_3_S_2_ ^[104]^ were used for the photocatalytic HER. Pollutants degradation with systems like bimetallic MoSrOS^[105]^ and V‐doped Mo(O,S)_2_ ^[106]^ were also reported. Among these systems, many could be good candidates for CO_2_ photoconversion. Especially MoCoOS that exhibits a Z‐scheme with appropriate band edges position for CO_2_ photoconversion. Indeed, the experimentally measured VB of Mo(O,S) (Mo oxysulfide: called “S‐doped Mo_4_O_11_” in Ref. [101]) is greater than the O_2_/H_2_O potential and the CB of Co(O,S) is way below the H_2_/H^+^ potential (Figure 6). Therefore, it would also be way above CO_2_ reduction potentials and hence be suitable for CO_2_ photoconversion from a thermodynamic point of view. At this stage, it is important to stress that the experimental CB/VB positions assigned to the synthesized Mo‐oxysulfide material illustrated in Figure 6, significantly differ from the theoretical ones assigned to similar materials in Figure 5. This trend is also valid for the positions of the CB/VB edges of α‐MoO_3_ which positions are shifted to more negative potentials.[^101^, ^107^] Possible origins of these shifts are provided in literature:^[108]^ the reduction of the pristine α‐MoO_3_ into substoichiometric MoO_x_ material and the hydroxylation of the α‐MoO_3_ surfaces. Interestingly similar effects have been reported for TiO_2_ surfaces.[^73^, ^109^] As a consequence, it is highly crucial to precisely control the surface state of the Mo‐oxysulfides (S/O ratio, surface hydroxyl or sulfhydryl groups…) in order to reach the targeted properties.

Even though transition metal oxysulfides are not widely reported in literature for photocatalytic applications, they exhibit interesting properties for this application and should not be forgotten when developing new photocatalysts. The heterojunction of Mo oxysulfides with TiO_2_ seems to be a particularly appealing strategy for CO_2_ photoconversion. Moreover, the genesis of such metal oxysulfides from an oxide precursor supported on TiO_2_ would require the use of sulfiding agent. This step may simultaneously induce the S‐doping of bulk TiO_2_ or TiO_2_ surfaces which has been shown to reduce the bandgap and shift the light absorption of TiO_2_ from UV to visible light.[^73^, ^110^, ^111^] As a consequence, a synergy effect for TiO_2_ supported metal oxysulfides can be expected at various levels: from light absorption to reactivity.

## 5 Conclusions and Perspectives

Even though the photoreduction of CO_2_ is a hot topic in the literature, it remains a daunting task to reach high enough conversion yields for industrialization perspectives (10 % for at least 10 years[^10^, ^11^]) with this approach. Challenges currently encompass both process and materials aspects so as to reach high efficiencies, sustainability and economic feasibility. Indeed, multiple operating parameters (reactor type, temperature, pressure, reactants type, light intensity, etc.) greatly affect the overall process efficiency as well as key materials properties (bandgap, band positions, charges conductivity, affinity with CO_2_, etc.). Up to date, the best systems record energetic efficiencies of around 1 % and often use scarce materials. They are hence not ready for industrialization and use at a large scale. Therefore, the search of efficient and sustainable photocatalysts to convert CO_2_ remains as one of nowadays biggest challenges.

TiO_2_ is a widely studied photocatalysts which shows serious drawbacks to be a good candidate for CO_2_ conversion by itself. Several strategies have been reported to improve its activity, the most efficient being the use of a Pt co‐catalyst^[13]^ which cannot be proposed as an industrial solution due to the serious constraints on this strategic and critical metal. MoS_2_ which is rather known for its good electrocatalytic properties can also be used as a photocatalyst even though it suffers from stability and overpotentials limitations. However, it has been reported as a better TiO_2_ co‐catalyst than Pt for H_2_ production^[69]^ and has hence great potential for CO_2_ photoreduction. Indeed, few articles, that were mentioned in this perspective, with promising results report a MoS_2_/TiO_2_ heterojunction for CO_2_ photocatalytic reduction with good improvement compared to bare TiO_2_.

However, the MoS_2_/TiO_2_ system may not be sufficient to reach high efficiency and within this perspective, we suggest to explore more deeply Mo oxysulfides supported on TiO_2_, which may offer more favorable properties for CO_2_ photoreduction. In this perspective oxysulfides advantages were highlighted: easy bandgap and band positions tuning depending on the chemical composition of the oxysulfide,[^95^, ^98^] stability toward photo‐oxidation,^[96]^ and favorable CO_2_ adsorption.^[100]^ Moreover, other wide bandgap semiconductors than TiO_2_, like CdS, WO_3_, ZnO, etc. could be combined to Mo oxysulfides, providing their band positions are appropriate to provide good heterojunction schemes.

Considering the analysis provided in this Perspective, it seems particularly interesting to encourage more systematic synthesis, characterization and photocatalytic evaluation of Mo oxysulfide materials for the challenging CO_2_ photoreduction reaction, which has not been done yet. Only few applications for H_2_ production and pollutants degradation have been reported.

## Conflict of Interests

The authors declare no conflict of interest.
